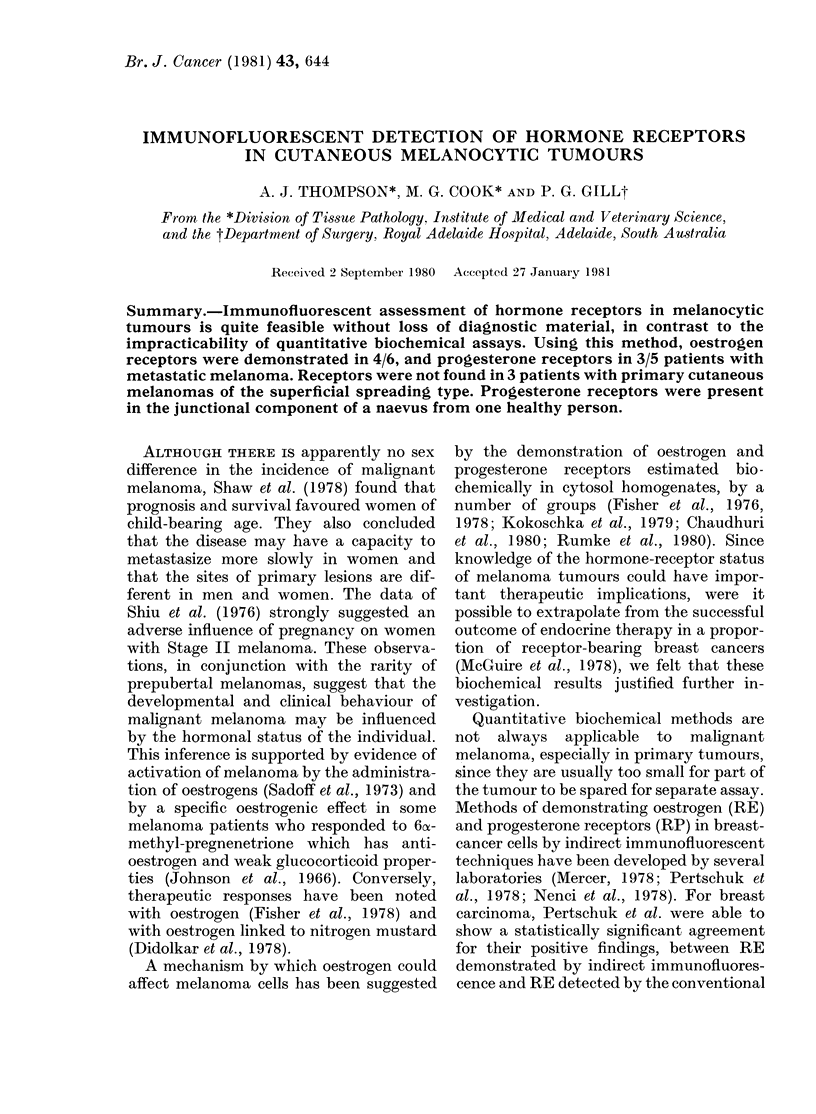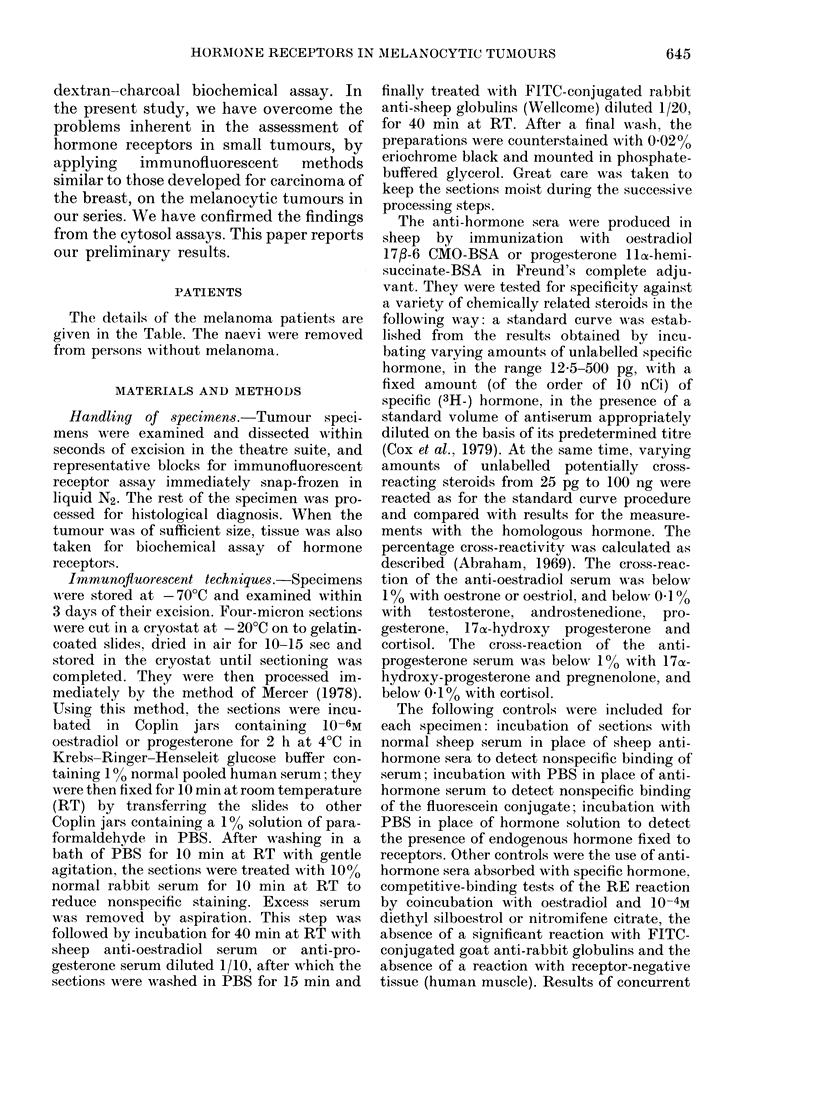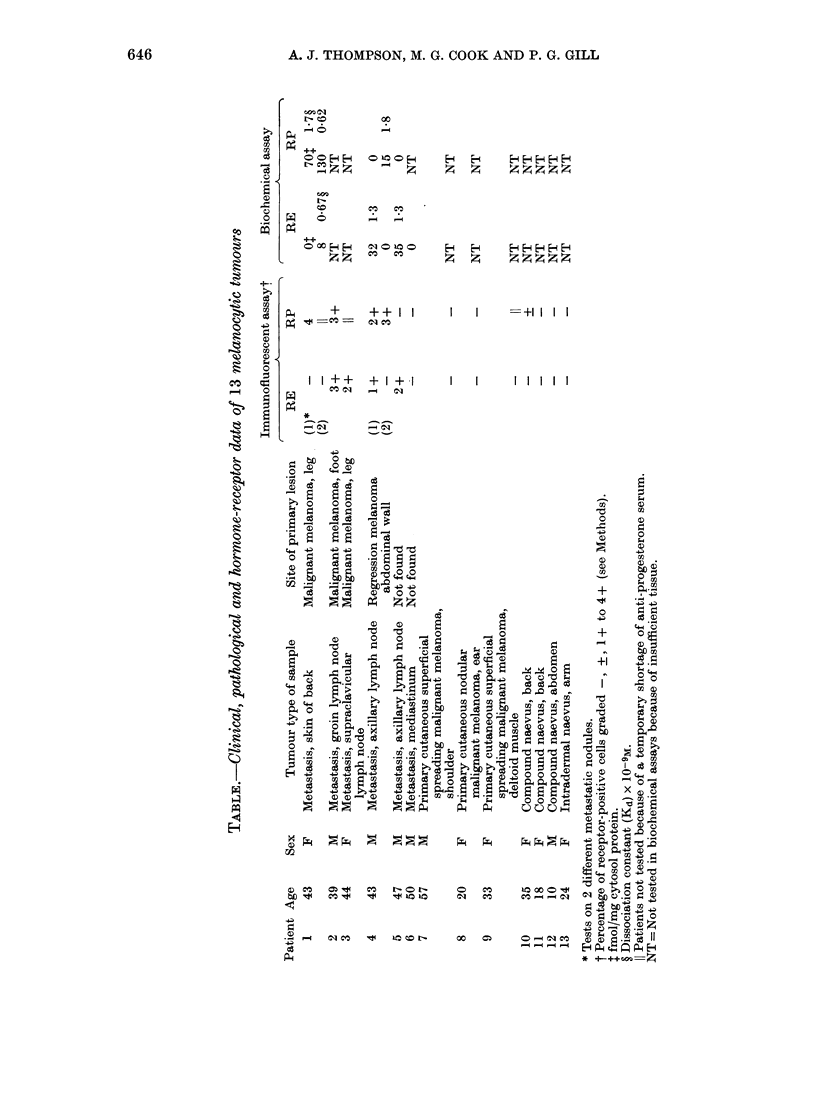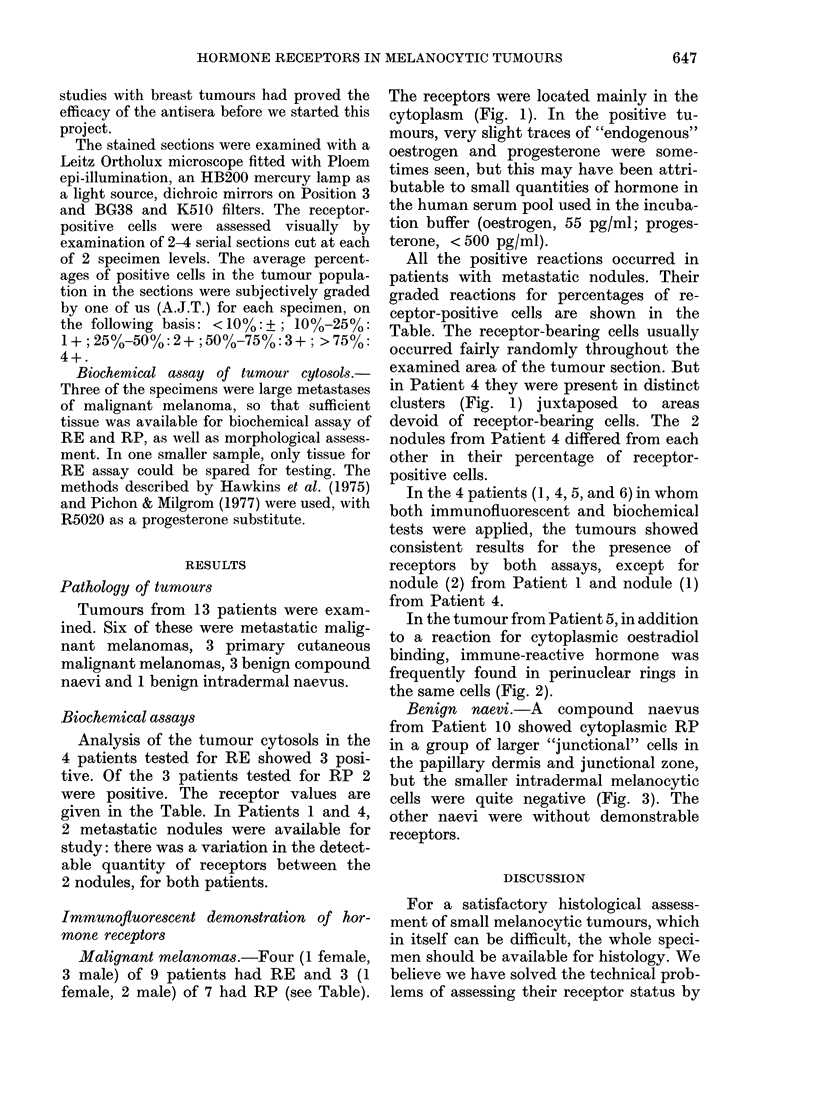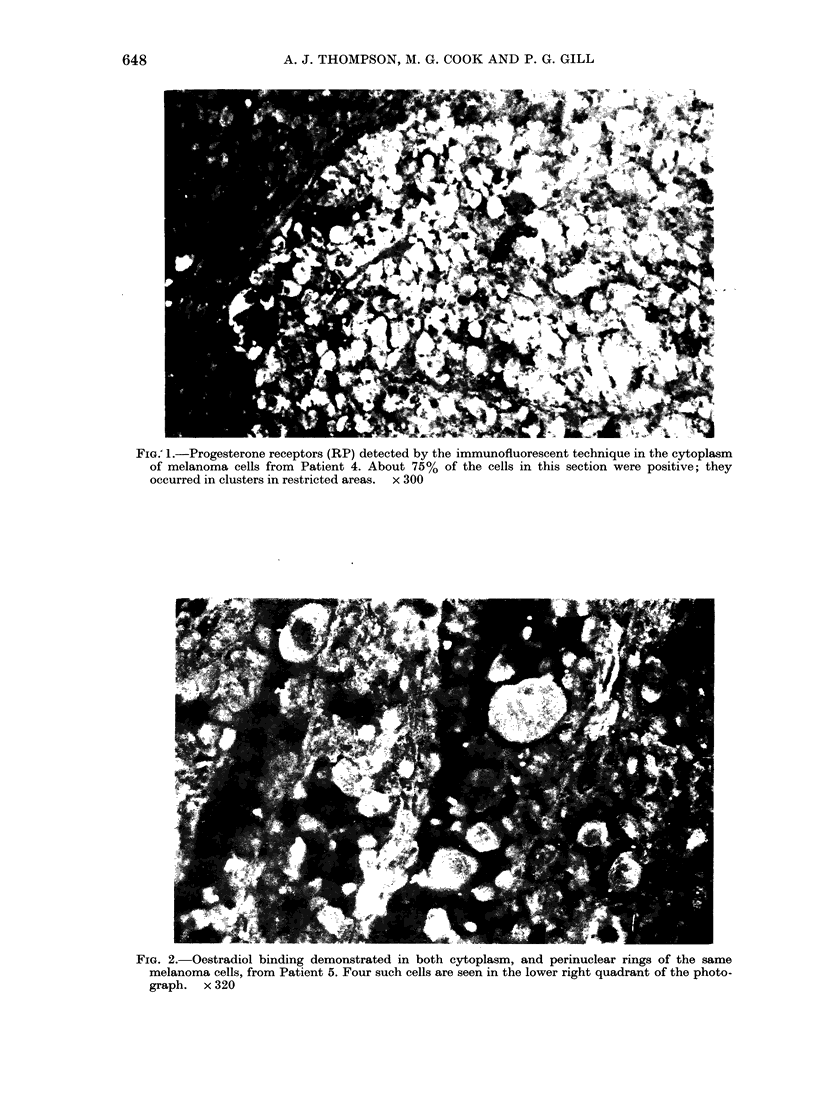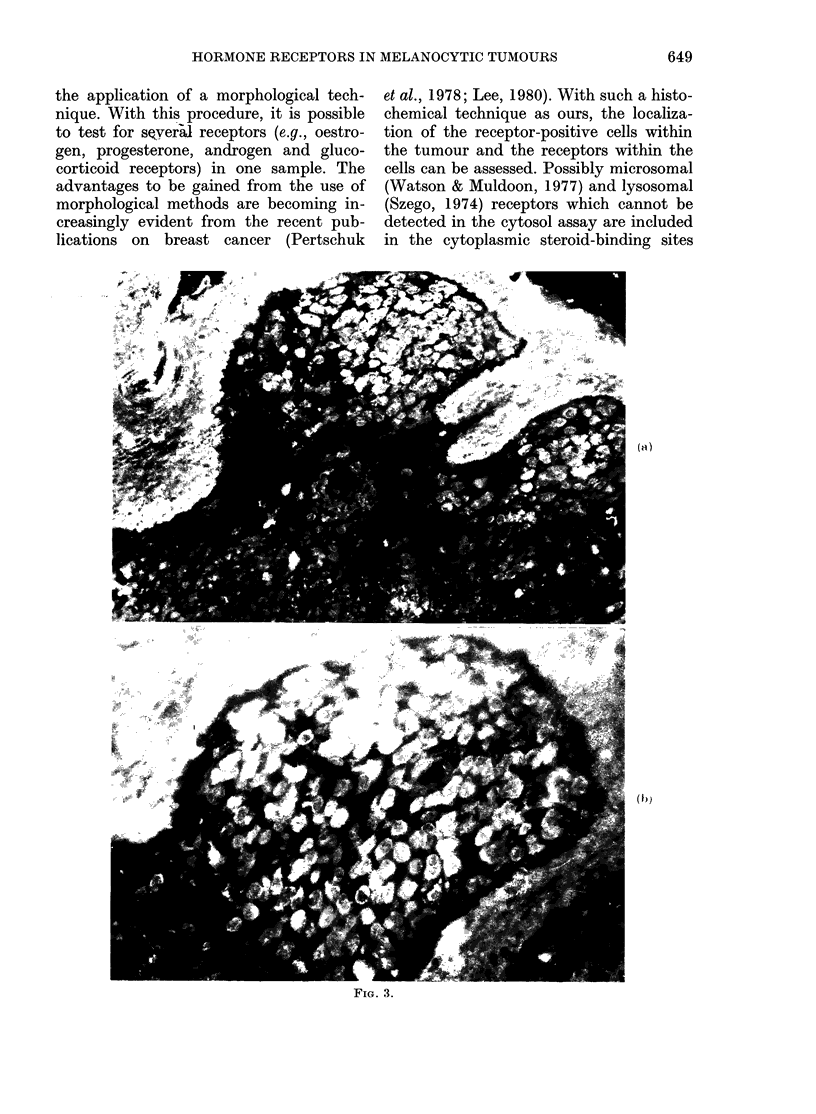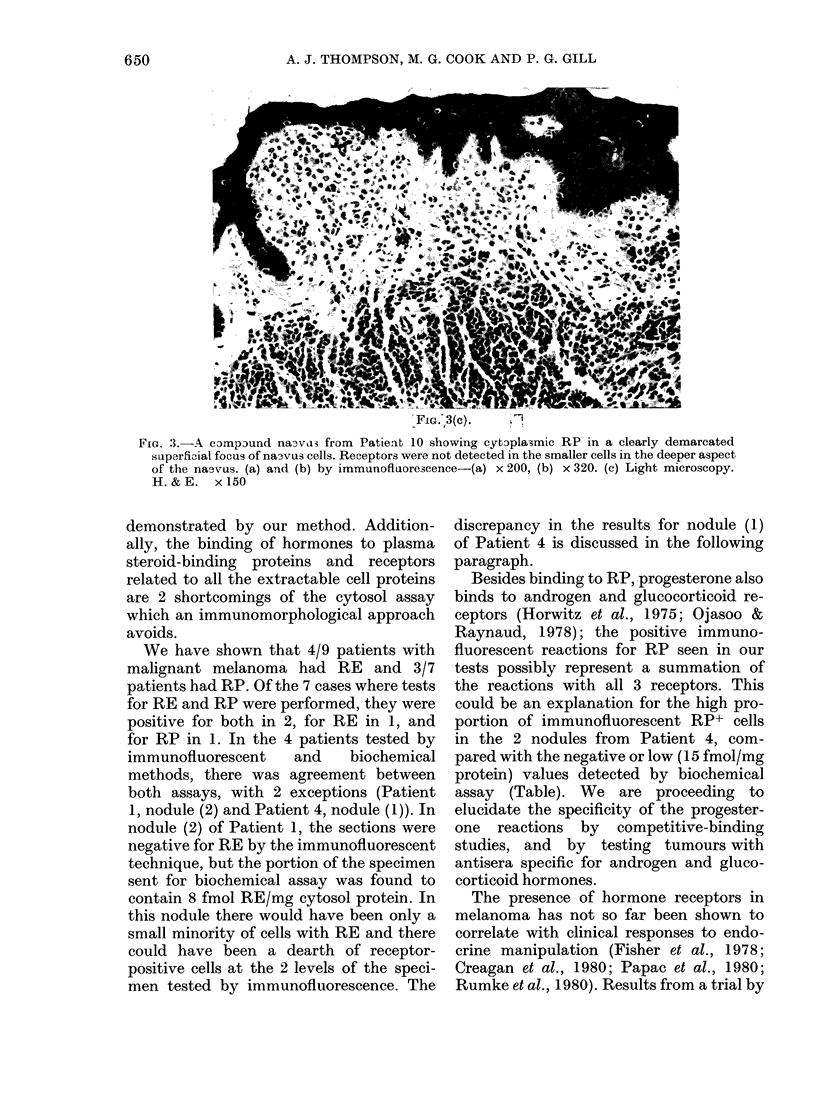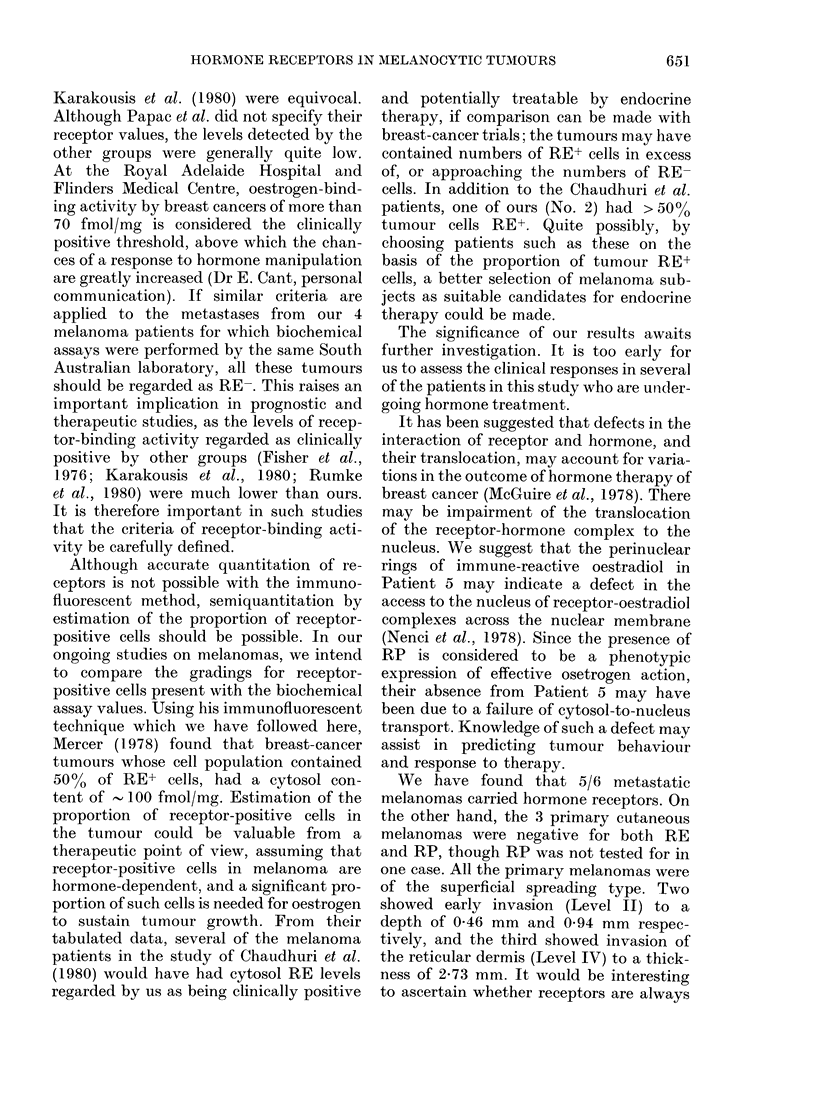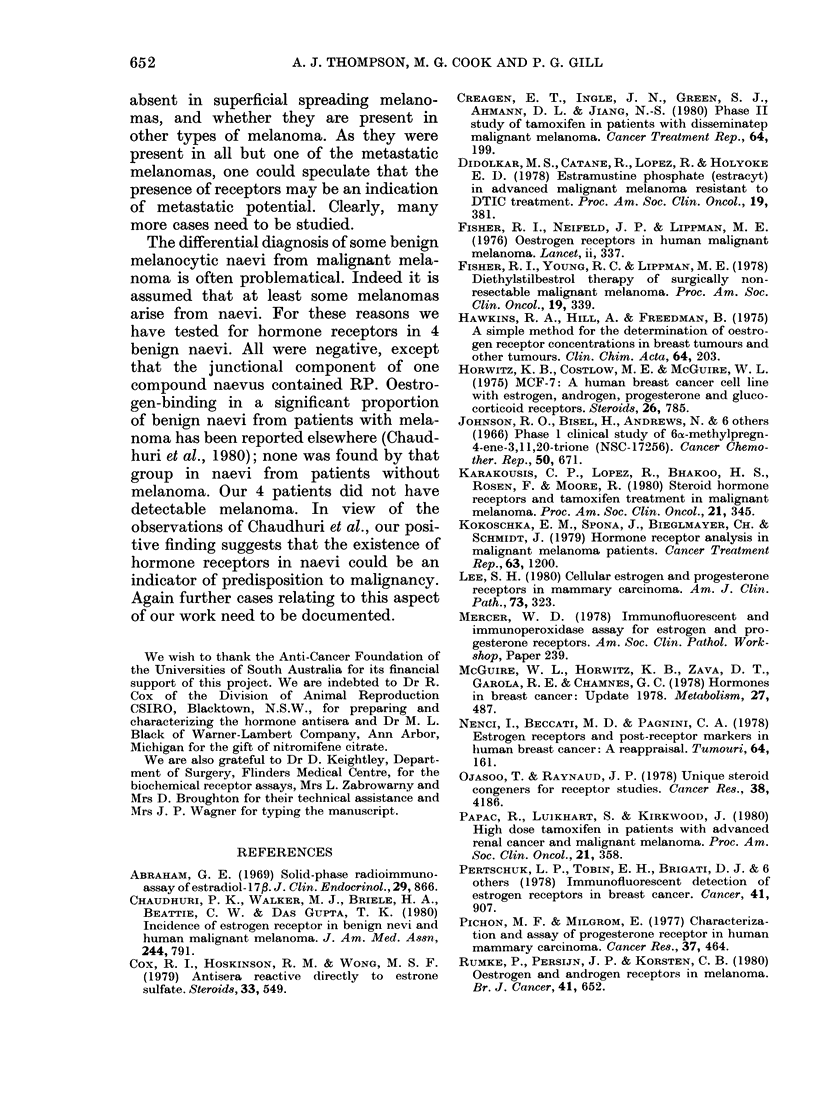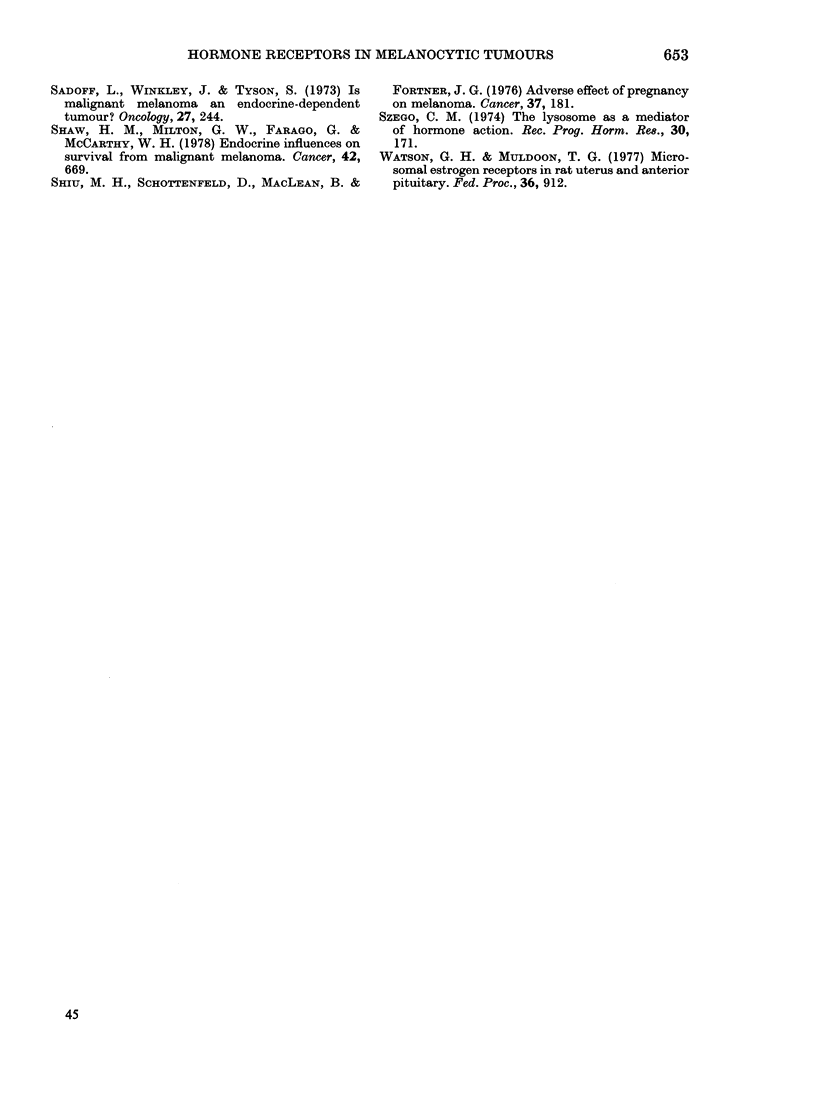# Immunofluorescent detection of hormone receptors in cutaneous melanocytic tumours.

**DOI:** 10.1038/bjc.1981.95

**Published:** 1981-05

**Authors:** A. J. Thompson, M. G. Cook, P. G. Gill

## Abstract

**Images:**


Br. J. Cancer (1981) 43, 644

### IMMUNOFLUORESCENT DETECTION OF HORMONE RECEPTORS IN CUTANEOUS MELANOCYTIC TUMOURS

A. J. THOMPSON*, M. G. COOK* AND P. G. GILL†

*From the *Division of Tissue Pathology, Institute of Medical and Veterinary Science, and the †Department of Surgery, Royal Adelaide Hospital, Adelaide, South Australia*

Received 2 September 1980 Accepted 27 January 1981

**Summary.—Immunofluorescent assessment of hormone receptors in melanocytic tumours is quite feasible without loss of diagnostic material, in contrast to the impracticability of quantitative biochemical assays. Using this method, oestrogen receptors were demonstrated in 4/6, and progesterone receptors in 3/5 patients with metastatic melanoma. Receptors were not found in 3 patients with primary cutaneous melanomas of the superficial spreading type. Progesterone receptors were present in the junctional component of a naevus from one healthy person.**

ALTHOUGH THERE IS apparently no sex difference in the incidence of malignant melanoma, Shaw *et al.* (1978) found that prognosis and survival favoured women of child-bearing age. They also concluded that the disease may have a capacity to metastasize more slowly in women and that the sites of primary lesions are different in men and women. The data of Shiu *et al.* (1976) strongly suggested an adverse influence of pregnancy on women with Stage II melanoma. These observations, in conjunction with the rarity of prepubertal melanomas, suggest that the developmental and clinical behaviour of malignant melanoma may be influenced by the hormonal status of the individual. This inference is supported by evidence of activation of melanoma by the administration of oestrogens (Sadoff *et al.*, 1973) and by a specific oestrogenic effect in some melanoma patients who responded to 6α-methyl-pregnenetrione which has anti-oestrogen and weak glucocorticoid properties (Johnson *et al.*, 1966). Conversely, therapeutic responses have been noted with oestrogen (Fisher *et al.*, 1978) and with oestrogen linked to nitrogen mustard (Didolkar *et al.*, 1978).

A mechanism by which oestrogen could affect melanoma cells has been suggested by the demonstration of oestrogen and progesterone receptors estimated biochemically in cytosol homogenates, by a number of groups (Fisher *et al.*, 1976, 1978; Kokoschka *et al.*, 1979; Chaudhuri *et al.*, 1980; Rumke *et al.*, 1980). Since knowledge of the hormone-receptor status of melanoma tumours could have important therapeutic implications, were it possible to extrapolate from the successful outcome of endocrine therapy in a proportion of receptor-bearing breast cancers (McGuire *et al.*, 1978), we felt that these biochemical results justified further investigation.

Quantitative biochemical methods are not always applicable to malignant melanoma, especially in primary tumours, since they are usually too small for part of the tumour to be spared for separate assay. Methods of demonstrating oestrogen (RE) and progesterone receptors (RP) in breast-cancer cells by indirect immunofluorescent techniques have been developed by several laboratories (Mercer, 1978; Pertschuk *et al.*, 1978; Nenci *et al.*, 1978). For breast carcinoma, Pertschuk *et al.* were able to show a statistically significant agreement for their positive findings, between RE demonstrated by indirect immunofluorescence and RE detected by the conventional dextran–charcoal biochemical assay. In the present study, we have overcome the problems inherent in the assessment of hormone receptors in small tumours, by applying immunofluorescent methods similar to those developed for carcinoma of the breast, on the melanocytic tumours in our series. We have confirmed the findings from the cytosol assays. This paper reports our preliminary results.

### PATIENTS

The details of the melanoma patients are given in the Table. The naevi were removed from persons without melanoma.

### MATERIALS AND METHODS

*Handling of specimens.*—Tumour specimens were examined and dissected within seconds of excision in the theatre suite, and representative blocks for immunofluorescent receptor assay immediately snap-frozen in liquid $N_2$. The rest of the specimen was processed for histological diagnosis. When the tumour was of sufficient size, tissue was also taken for biochemical assay of hormone receptors.

*Immunofluorescent techniques.*—Specimens were stored at −70°C and examined within 3 days of their excision. Four-micron sections were cut in a cryostat at −20°C on to gelatin-coated slides, dried in air for 10–15 sec and stored in the cryostat until sectioning was completed. They were then processed immediately by the method of Mercer (1978). Using this method, the sections were incubated in Coplin jars containing $10^{-6}$M oestradiol or progesterone for 2 h at 4°C in Krebs–Ringer–Henseleit glucose buffer containing 1% normal pooled human serum; they were then fixed for 10 min at room temperature (RT) by transferring the slides to other Coplin jars containing a 1% solution of paraformaldehyde in PBS. After washing in a bath of PBS for 10 min at RT with gentle agitation, the sections were treated with 10% normal rabbit serum for 10 min at RT to reduce nonspecific staining. Excess serum was removed by aspiration. This step was followed by incubation for 40 min at RT with sheep anti-oestradiol serum or anti-progesterone serum diluted 1/10, after which the sections were washed in PBS for 15 min and finally treated with FITC-conjugated rabbit anti-sheep globulins (Wellcome) diluted 1/20, for 40 min at RT. After a final wash, the preparations were counterstained with 0·02% eriochrome black and mounted in phosphate-buffered glycerol. Great care was taken to keep the sections moist during the successive processing steps.

The anti-hormone sera were produced in sheep by immunization with oestradiol 17β-6 CMO-BSA or progesterone 11α-hemisuccinate-BSA in Freund's complete adjuvant. They were tested for specificity against a variety of chemically related steroids in the following way: a standard curve was established from the results obtained by incubating varying amounts of unlabelled specific hormone, in the range 12·5–500 pg, with a fixed amount (of the order of 10 nCi) of specific ($^3$H-) hormone, in the presence of a standard volume of antiserum appropriately diluted on the basis of its predetermined titre (Cox *et al.*, 1979). At the same time, varying amounts of unlabelled potentially cross-reacting steroids from 25 pg to 100 ng were reacted as for the standard curve procedure and compared with results for the measurements with the homologous hormone. The percentage cross-reactivity was calculated as described (Abraham, 1969). The cross-reaction of the anti-oestradiol serum was below 1% with oestrone or oestriol, and below 0·1% with testosterone, androstenedione, progesterone, 17α-hydroxy progesterone and cortisol. The cross-reaction of the anti-progesterone serum was below 1% with 17α-hydroxy-progesterone and pregnenolone, and below 0·1% with cortisol.

The following controls were included for each specimen: incubation of sections with normal sheep serum in place of sheep anti-hormone sera to detect nonspecific binding of serum; incubation with PBS in place of anti-hormone serum to detect nonspecific binding of the fluorescein conjugate; incubation with PBS in place of hormone solution to detect the presence of endogenous hormone fixed to receptors. Other controls were the use of anti-hormone sera absorbed with specific hormone, competitive-binding tests of the RE reaction by coincubation with oestradiol and $10^{-4}$M diethyl silboestrol or nitromifene citrate, the absence of a significant reaction with FITC-conjugated goat anti-rabbit globulins and the absence of a reaction with receptor-negative tissue (human muscle). Results of concurrent

TABLE.—*Clinical, pathological and hormone-receptor data of 13 melanocytic tumours*

| Patient | Age | Sex | Tumour type of sample | Site of primary lesion | | Immunofluorescent assay† RE | Immunofluorescent assay† RP | Biochemical assay RE | | Biochemical assay RP | |
|---|---|---|---|---|---|---|---|---|---|---|---|
| 1 | 43 | F | Metastasis, skin of back | Malignant melanoma, leg | (1)* | – | 4 | 0‡ | | 70‡ | 1·7§ |
| | | | | | (2) | – | ‖ | 8 | 0·67§ | 130 | 0·62 |
| 2 | 39 | M | Metastasis, groin lymph node | Malignant melanoma, foot | | 3+ | 3+ | NT | | NT | |
| 3 | 44 | F | Metastasis, supraclavicular lymph node | Malignant melanoma, leg | | 2+ | ‖ | NT | | NT | |
| 4 | 43 | M | Metastasis, axillary lymph node | Regression melanoma abdominal wall | (1) | 1+ | 2+ | 32 | 1·3 | 0 | |
| | | | | | (2) | – | 3+ | 0 | | 15 | 1·8 |
| 5 | 47 | M | Metastasis, axillary lymph node | Not found | | 2+ | – | 35 | 1·3 | 0 | |
| 6 | 50 | M | Metastasis, mediastinum | Not found | | – | – | 0 | | NT | |
| 7 | 57 | M | Primary cutaneous superficial spreading malignant melanoma, shoulder | | | – | – | NT | | NT | |
| 8 | 20 | F | Primary cutaneous nodular malignant melanoma, ear | | | – | – | NT | | NT | |
| 9 | 33 | F | Primary cutaneous superficial spreading malignant melanoma, deltoid muscle | | | – | ‖ | NT | | NT | |
| 10 | 35 | F | Compound naevus, back | | | – | ± | NT | | NT | |
| 11 | 18 | F | Compound naevus, back | | | – | – | NT | | NT | |
| 12 | 10 | M | Compound naevus, abdomen | | | – | – | NT | | NT | |
| 13 | 24 | F | Intradermal naevus, arm | | | – | – | NT | | NT | |

* Tests on 2 different metastatic nodules.

† Percentage of receptor-positive cells graded –, ±, 1+ to 4+ (see Methods).

‡ fmol/mg cytosol protein.

§ Dissociation constant ($K_d$) × $10^{-9}$M.

‖ Patients not tested because of a temporary shortage of anti-progesterone serum.

NT = Not tested in biochemical assays because of insufficient tissue.

studies with breast tumours had proved the efficacy of the antisera before we started this project.

The stained sections were examined with a Leitz Ortholux microscope fitted with Ploem epi-illumination, an HB200 mercury lamp as a light source, dichroic mirrors on Position 3 and BG38 and K510 filters. The receptor-positive cells were assessed visually by examination of 2–4 serial sections cut at each of 2 specimen levels. The average percentages of positive cells in the tumour population in the sections were subjectively graded by one of us (A.J.T.) for each specimen, on the following basis: <10%: ±; 10%–25%: 1+; 25%–50%: 2+; 50%–75%: 3+; >75%: 4+.

*Biochemical assay of tumour cytosols.*—Three of the specimens were large metastases of malignant melanoma, so that sufficient tissue was available for biochemical assay of RE and RP, as well as morphological assessment. In one smaller sample, only tissue for RE assay could be spared for testing. The methods described by Hawkins *et al.* (1975) and Pichon & Milgrom (1977) were used, with R5020 as a progesterone substitute.

### RESULTS

### *Pathology of tumours*

Tumours from 13 patients were examined. Six of these were metastatic malignant melanomas, 3 primary cutaneous malignant melanomas, 3 benign compound naevi and 1 benign intradermal naevus.

### *Biochemical assays*

Analysis of the tumour cytosols in the 4 patients tested for RE showed 3 positive. Of the 3 patients tested for RP 2 were positive. The receptor values are given in the Table. In Patients 1 and 4, 2 metastatic nodules were available for study: there was a variation in the detectable quantity of receptors between the 2 nodules, for both patients.

### *Immunofluorescent demonstration of hormone receptors*

*Malignant melanomas.*—Four (1 female, 3 male) of 9 patients had RE and 3 (1 female, 2 male) of 7 had RP (see Table). The receptors were located mainly in the cytoplasm (Fig. 1). In the positive tumours, very slight traces of "endogenous" oestrogen and progesterone were sometimes seen, but this may have been attributable to small quantities of hormone in the human serum pool used in the incubation buffer (oestrogen, 55 pg/ml; progesterone, < 500 pg/ml).

All the positive reactions occurred in patients with metastatic nodules. Their graded reactions for percentages of receptor-positive cells are shown in the Table. The receptor-bearing cells usually occurred fairly randomly throughout the examined area of the tumour section. But in Patient 4 they were present in distinct clusters (Fig. 1) juxtaposed to areas devoid of receptor-bearing cells. The 2 nodules from Patient 4 differed from each other in their percentage of receptor-positive cells.

In the 4 patients (1, 4, 5, and 6) in whom both immunofluorescent and biochemical tests were applied, the tumours showed consistent results for the presence of receptors by both assays, except for nodule (2) from Patient 1 and nodule (1) from Patient 4.

In the tumour from Patient 5, in addition to a reaction for cytoplasmic oestradiol binding, immune-reactive hormone was frequently found in perinuclear rings in the same cells (Fig. 2).

*Benign naevi.*—A compound naevus from Patient 10 showed cytoplasmic RP in a group of larger "junctional" cells in the papillary dermis and junctional zone, but the smaller intradermal melanocytic cells were quite negative (Fig. 3). The other naevi were without demonstrable receptors.

### DISCUSSION

For a satisfactory histological assessment of small melanocytic tumours, which in itself can be difficult, the whole specimen should be available for histology. We believe we have solved the technical problems of assessing their receptor status by

FIG. 1.—Progesterone receptors (RP) detected by the immunofluorescent technique in the cytoplasm of melanoma cells from Patient 4. About 75% of the cells in this section were positive; they occurred in clusters in restricted areas. ×300

FIG. 2.—Oestradiol binding demonstrated in both cytoplasm, and perinuclear rings of the same melanoma cells, from Patient 5. Four such cells are seen in the lower right quadrant of the photograph. ×320

the application of a morphological technique. With this procedure, it is possible to test for several receptors (*e.g.*, oestrogen, progesterone, androgen and glucocorticoid receptors) in one sample. The advantages to be gained from the use of morphological methods are becoming increasingly evident from the recent publications on breast cancer (Pertschuk *et al.*, 1978; Lee, 1980). With such a histochemical technique as ours, the localization of the receptor-positive cells within the tumour and the receptors within the cells can be assessed. Possibly microsomal (Watson & Muldoon, 1977) and lysosomal (Szego, 1974) receptors which cannot be detected in the cytosol assay are included in the cytoplasmic steroid-binding sites

(a)
(b)

FIG. 3.

FIG. 3(c).

FIG. 3.—A compound naevus from Patient 10 showing cytoplasmic RP in a clearly demarcated superficial focus of naevus cells. Receptors were not detected in the smaller cells in the deeper aspect of the naevus. (a) and (b) by immunofluorescence—(a) ×200, (b) ×320. (c) Light microscopy. H. & E. ×150

demonstrated by our method. Additionally, the binding of hormones to plasma steroid-binding proteins and receptors related to all the extractable cell proteins are 2 shortcomings of the cytosol assay which an immunomorphological approach avoids.

We have shown that 4/9 patients with malignant melanoma had RE and 3/7 patients had RP. Of the 7 cases where tests for RE and RP were performed, they were positive for both in 2, for RE in 1, and for RP in 1. In the 4 patients tested by immunofluorescent and biochemical methods, there was agreement between both assays, with 2 exceptions (Patient 1, nodule (2) and Patient 4, nodule (1)). In nodule (2) of Patient 1, the sections were negative for RE by the immunofluorescent technique, but the portion of the specimen sent for biochemical assay was found to contain 8 fmol RE/mg cytosol protein. In this nodule there would have been only a small minority of cells with RE and there could have been a dearth of receptor-positive cells at the 2 levels of the specimen tested by immunofluorescence. The discrepancy in the results for nodule (1) of Patient 4 is discussed in the following paragraph.

Besides binding to RP, progesterone also binds to androgen and glucocorticoid receptors (Horwitz *et al.*, 1975; Ojasoo & Raynaud, 1978); the positive immunofluorescent reactions for RP seen in our tests possibly represent a summation of the reactions with all 3 receptors. This could be an explanation for the high proportion of immunofluorescent $RP^+$ cells in the 2 nodules from Patient 4, compared with the negative or low (15 fmol/mg protein) values detected by biochemical assay (Table). We are proceeding to elucidate the specificity of the progesterone reactions by competitive-binding studies, and by testing tumours with antisera specific for androgen and glucocorticoid hormones.

The presence of hormone receptors in melanoma has not so far been shown to correlate with clinical responses to endocrine manipulation (Fisher *et al.*, 1978; Creagan *et al.*, 1980; Papac *et al.*, 1980; Rumke *et al.*, 1980). Results from a trial by

Karakousis *et al.* (1980) were equivocal. Although Papac *et al.* did not specify their receptor values, the levels detected by the other groups were generally quite low. At the Royal Adelaide Hospital and Flinders Medical Centre, oestrogen-binding activity by breast cancers of more than 70 fmol/mg is considered the clinically positive threshold, above which the chances of a response to hormone manipulation are greatly increased (Dr E. Cant, personal communication). If similar criteria are applied to the metastases from our 4 melanoma patients for which biochemical assays were performed by the same South Australian laboratory, all these tumours should be regarded as $RE^-$. This raises an important implication in prognostic and therapeutic studies, as the levels of receptor-binding activity regarded as clinically positive by other groups (Fisher *et al.*, 1976; Karakousis *et al.*, 1980; Rumke *et al.*, 1980) were much lower than ours. It is therefore important in such studies that the criteria of receptor-binding activity be carefully defined.

Although accurate quantitation of receptors is not possible with the immunofluorescent method, semiquantitation by estimation of the proportion of receptor-positive cells should be possible. In our ongoing studies on melanomas, we intend to compare the gradings for receptor-positive cells present with the biochemical assay values. Using his immunofluorescent technique which we have followed here, Mercer (1978) found that breast-cancer tumours whose cell population contained 50% of $RE^+$ cells, had a cytosol content of $\sim 100$ fmol/mg. Estimation of the proportion of receptor-positive cells in the tumour could be valuable from a therapeutic point of view, assuming that receptor-positive cells in melanoma are hormone-dependent, and a significant proportion of such cells is needed for oestrogen to sustain tumour growth. From their tabulated data, several of the melanoma patients in the study of Chaudhuri *et al.* (1980) would have had cytosol RE levels regarded by us as being clinically positive and potentially treatable by endocrine therapy, if comparison can be made with breast-cancer trials; the tumours may have contained numbers of $RE^+$ cells in excess of, or approaching the numbers of $RE^-$ cells. In addition to the Chaudhuri *et al.* patients, one of ours (No. 2) had $>50\%$ tumour cells $RE^+$. Quite possibly, by choosing patients such as these on the basis of the proportion of tumour $RE^+$ cells, a better selection of melanoma subjects as suitable candidates for endocrine therapy could be made.

The significance of our results awaits further investigation. It is too early for us to assess the clinical responses in several of the patients in this study who are undergoing hormone treatment.

It has been suggested that defects in the interaction of receptor and hormone, and their translocation, may account for variations in the outcome of hormone therapy of breast cancer (McGuire *et al.*, 1978). There may be impairment of the translocation of the receptor-hormone complex to the nucleus. We suggest that the perinuclear rings of immune-reactive oestradiol in Patient 5 may indicate a defect in the access to the nucleus of receptor-oestradiol complexes across the nuclear membrane (Nenci *et al.*, 1978). Since the presence of RP is considered to be a phenotypic expression of effective osetrogen action, their absence from Patient 5 may have been due to a failure of cytosol-to-nucleus transport. Knowledge of such a defect may assist in predicting tumour behaviour and response to therapy.

We have found that 5/6 metastatic melanomas carried hormone receptors. On the other hand, the 3 primary cutaneous melanomas were negative for both RE and RP, though RP was not tested for in one case. All the primary melanomas were of the superficial spreading type. Two showed early invasion (Level II) to a depth of 0·46 mm and 0·94 mm respectively, and the third showed invasion of the reticular dermis (Level IV) to a thickness of 2·73 mm. It would be interesting to ascertain whether receptors are always absent in superficial spreading melanomas, and whether they are present in other types of melanoma. As they were present in all but one of the metastatic melanomas, one could speculate that the presence of receptors may be an indication of metastatic potential. Clearly, many more cases need to be studied.

The differential diagnosis of some benign melanocytic naevi from malignant melanoma is often problematical. Indeed it is assumed that at least some melanomas arise from naevi. For these reasons we have tested for hormone receptors in 4 benign naevi. All were negative, except that the junctional component of one compound naevus contained RP. Oestrogen-binding in a significant proportion of benign naevi from patients with melanoma has been reported elsewhere (Chaudhuri *et al.*, 1980); none was found by that group in naevi from patients without melanoma. Our 4 patients did not have detectable melanoma. In view of the observations of Chaudhuri *et al.*, our positive finding suggests that the existence of hormone receptors in naevi could be an indicator of predisposition to malignancy. Again further cases relating to this aspect of our work need to be documented.

We wish to thank the Anti-Cancer Foundation of the Universities of South Australia for its financial support of this project. We are indebted to Dr R. Cox of the Division of Animal Reproduction CSIRO, Blacktown, N.S.W., for preparing and characterizing the hormone antisera and Dr M. L. Black of Warner-Lambert Company, Ann Arbor, Michigan for the gift of nitromifene citrate.

We are also grateful to Dr D. Keightley, Department of Surgery, Flinders Medical Centre, for the biochemical receptor assays, Mrs L. Zabrowarny and Mrs D. Broughton for their technical assistance and Mrs J. P. Wagner for typing the manuscript.

### REFERENCES

ABRAHAM, G. E. (1969) Solid-phase radioimmunoassay of estradiol-17$\beta$. *J. Clin. Endocrinol.*, **29**, 866.

CHAUDHURI, P. K., WALKER, M. J., BRIELE, H. A., BEATTIE, C. W. & DAS GUPTA, T. K. (1980) Incidence of estrogen receptor in benign nevi and human malignant melanoma. *J. Am. Med. Assn*, **244**, 791.

COX, R. I., HOSKINSON, R. M. & WONG, M. S. F. (1979) Antisera reactive directly to estrone sulfate. *Steroids*, **33**, 549.

CREAGEN, E. T., INGLE, J. N., GREEN, S. J., AHMANN, D. L. & JIANG, N.-S. (1980) Phase II study of tamoxifen in patients with disseminatep malignant melanoma. *Cancer Treatment Rep.*, **64**, 199.

DIDOLKAR, M. S., CATANE, R., LOPEZ, R. & HOLYOKE E. D. (1978) Estramustine phosphate (estracyt) in advanced malignant melanoma resistant to DTIC treatment. *Proc. Am. Soc. Clin. Oncol.*, **19**, 381.

FISHER, R. I., NEIFELD, J. P. & LIPPMAN, M. E. (1976) Oestrogen receptors in human malignant melanoma. *Lancet*, ii, 337.

FISHER, R. I., YOUNG, R. C. & LIPPMAN, M. E. (1978) Diethylstilbestrol therapy of surgically non-resectable malignant melanoma. *Proc. Am. Soc. Clin. Oncol.*, **19**, 339.

HAWKINS, R. A., HILL, A. & FREEDMAN, B. (1975) A simple method for the determination of oestrogen receptor concentrations in breast tumours and other tumours. *Clin. Chim. Acta*, **64**, 203.

HORWITZ, K. B., COSTLOW, M. E. & MCGUIRE, W. L. (1975) MCF-7: A human breast cancer cell line with estrogen, androgen, progesterone and glucocorticoid receptors. *Steroids*, **26**, 785.

JOHNSON, R. O., BISEL, H., ANDREWS, N. & 6 others (1966) Phase 1 clinical study of 6$\alpha$-methylpregn-4-ene-3,11,20-trione (NSC-17256). *Cancer Chemother. Rep.*, **50**, 671.

KARAKOUSIS, C. P., LOPEZ, R., BHAKOO, H. S., ROSEN, F. & MOORE, R. (1980) Steroid hormone receptors and tamoxifen treatment in malignant melanoma. *Proc. Am. Soc. Clin. Oncol.*, **21**, 345.

KOKOSCHKA, E. M., SPONA, J., BIEGLMAYER, CH. & SCHMIDT, J. (1979) Hormone receptor analysis in malignant melanoma patients. *Cancer Treatment Rep.*, **63**, 1200.

LEE, S. H. (1980) Cellular estrogen and progesterone receptors in mammary carcinoma. *Am. J. Clin. Path.*, **73**, 323.

MERCER, W. D. (1978) Immunofluorescent and immunoperoxidase assay for estrogen and progesterone receptors. *Am. Soc. Clin. Pathol. Workshop*, Paper 239.

MCGUIRE, W. L., HORWITZ, K. B., ZAVA, D. T., GAROLA, R. E. & CHAMNES, G. C. (1978) Hormones in breast cancer: Update 1978. *Metabolism*, **27**, 487.

NENCI, I., BECCATI, M. D. & PAGNINI, C. A. (1978) Estrogen receptors and post-receptor markers in human breast cancer: A reappraisal. *Tumouri*, **64**, 161.

OJASOO, T. & RAYNAUD, J. P. (1978) Unique steroid congeners for receptor studies. *Cancer Res.*, **38**, 4186.

PAPAC, R., LUIKHART, S. & KIRKWOOD, J. (1980) High dose tamoxifen in patients with advanced renal cancer and malignant melanoma. *Proc. Am. Soc. Clin. Oncol.*, **21**, 358.

PERTSCHUK, L. P., TOBIN, E. H., BRIGATI, D. J. & 6 others (1978) Immunofluorescent detection of estrogen receptors in breast cancer. *Cancer*, **41**, 907.

PICHON, M. F. & MILGROM, E. (1977) Characterization and assay of progesterone receptor in human mammary carcinoma. *Cancer Res.*, **37**, 464.

RUMKE, P., PERSIJN, J. P. & KORSTEN, C. B. (1980) Oestrogen and androgen receptors in melanoma. *Br. J. Cancer*, **41**, 652.

SADOFF, L., WINKLEY, J. & TYSON, S. (1973) Is malignant melanoma an endocrine-dependent tumour? *Oncology*, **27**, 244.
SHAW, H. M., MILTON, G. W., FARAGO, G. & MCCARTHY, W. H. (1978) Endocrine influences on survival from malignant melanoma. *Cancer*, **42**, 669.
SHIU, M. H., SCHOTTENFELD, D., MACLEAN, B. & FORTNER, J. G. (1976) Adverse effect of pregnancy on melanoma. *Cancer*, **37**, 181.
SZEGO, C. M. (1974) The lysosome as a mediator of hormone action. *Rec. Prog. Horm. Res.*, **30**, 171.
WATSON, G. H. & MULDOON, T. G. (1977) Microsomal estrogen receptors in rat uterus and anterior pituitary. *Fed. Proc.*, **36**, 912.